# CT–based AI score associates with perioperative outcomes in nephron–sparing surgery for renal cell carcinoma

**DOI:** 10.1186/s40644-025-00961-2

**Published:** 2025-12-29

**Authors:** Lin Shengfa, Su Liqing, Chen Shu, Chen Huijian, Lin Yuying, Lin Zijie, Xia Yinfeng, Li Qianwen, Fang Zhuting, Ma Mingping, Hu Minxiong

**Affiliations:** 1 Department of Radiology, Shengli Clinical Medical College of Fujian Medical University, Fujian Provincial Hospital, Fuzhou University Affiliated Provincial Hospital, No. 134, Dong Street, Gulou District, Fuzhou, Fujian 350001 China; 2 Department of Oncology and Vascular Intervention, Clinical Oncology School of Fujian Medical University, Fujian Cancer Hospital, No. 420, Fuma Road, Jinan District, Fuzhou, Fujian 350011 China; 3 Department of Radiology, Clinical Oncology School of Fujian Medical University, Fujian Cancer Hospital, Fuzhou, Fujian 350011 China; 4 College of Physics and Information Engineering, Fuzhou University, Fuzhou, Fujian 350001 China; 5 Information Management Center, Provincial Key Laboratory of Medical Big Data Engineering, Fuzhou University Affiliated Provincial Hospital, Fuzhou, Fujian 350001 China; 6 Department of Interventional Radiology, Shengli Clinical Medical College of Fujian Medical University, Fujian Provincial Hospital, Fuzhou University Affiliated Provincial Hospital, Fuzhou, Fujian 350001 China; 7 Department of Urology, Shengli Clinical Medical College of Fujian Medical University, Fuzhou University Affiliated Provincial Hospital, No. 134, Dong Street, Gulou District, Fuzhou, Fujian 350001 China

**Keywords:** Renal cell carcinoma, Computed tomography, Artificial intelligence, Nephron-sparing surgery, R.E.N.A.L. nephrometry, Contact surface area

## Abstract

**Background:**

To develop and validate a CT–based artificial intelligence (AI) score model integrating the R.E.N.A.L. nephrometry and contact surface area (CSA) for efficient, accurate prediction of perioperative outcomes in renal cell carcinoma (RCC) patients undergoing nephron–sparing surgery (NSS), addressing the subjectivity and inefficiency of manual score.

**Methods:**

Retrospectively collected data from two NSS cohorts (n1 = 500, n2 = 50): 90% of cases in Cohort n1 (450 cases) were randomly assigned to the training set (315 cases), validation set (45 cases), and test set (90 cases) at a ratio of 7:1:2, which were used to develop and validate the automated kidney/tumor segmentation models, as well as to derive the AI-calculated R.E.N.A.L. score (with the “A” parameter excluded) and AI-calculated CSA score; the remaining 10% of cases in Cohort n1 (50 cases) were combined with all 50 cases in Cohort n2 to form a mixed validation set (100 cases), which was used for risk stratification prediction of NSS perioperative outcomes via AI scores. Manual image annotation/scoring was conducted by experienced radiologists and urologists. Interrater consistency was evaluated via weighted kappa coefficients; risk stratification was performed via Kruskal–Wallis tests and Mann–Whitney U tests.

**Results:**

A total of 550 patients were included in this study (median age, 56 [IQR: 46–66] years; 341 males). The segmentation model exhibited excellent performance: Dice similarity coefficient (DSC) was 0.95 for kidneys and 0.80 for tumors; normalized surface distance (NSD) was 0.923 ± 0.082 and 0.892 ± 0.096, respectively; 95th percentile Hausdorff distance (HD95) was 9.78 ± 0.63 mm and 12.65 ± 0.84 mm, respectively. The R, E, N, L, R.E.N.A.L., and CSA score models had good consistency compared with the manual score, and the kappa coefficients were 0.82, 0.49, 0.63, 0.60, 0.65, and 0.69, respectively (all *P* < 0.01). Risk stratification by AI score significantly predicted warm ischemia time, surgical duration, intraoperative blood loss, serum creatinine changes, pathological T stage, and nuclear grade (all *P* < 0.05).

**Conclusions:**

This study successfully developed a CT-based automated kidney/tumor segmentation model, and on this basis constructed the AI-R.E.N.A.L. and AI-CSA scoring models, providing an efficient and objective preoperative risk assessment tool for the perioperative outcomes of NSS.

**Supplementary Information:**

The online version contains supplementary material available at 10.1186/s40644-025-00961-2.

## Introduction

Renal cell carcinoma (RCC) accounts for 2%–3% of adult malignant tumors, with a rising global incidence (average annual growth rate: 0.7%–2% over the past decade) [1–4]. Advances in imaging techniques (ultrasound, CT/MRI) have significantly improved early RCC detection [5]. Clinical guidelines designate nephron–sparing surgery (NSS) as the preferred treatment for T1a RCC and a viable option for selecting T1b tumors [6, 7]. Tumor anatomical characteristics, such as size, location, relationship to the renal vasculature/collecting system, and contact surface area (CSA), are pivotal determinants of NSS outcomes. Widely used scoring systems, including the R.E.N.A.L. nephrometry score [8], PADUA classification [9], and renal tumor CSA quantify these features from CT/MRI to independently predict perioperative outcomes such as warm ischemia time (WIT), operative duration, blood loss, pathological stage, and nuclear grade [10]. Among them, the R.E.N.A.L. score includes five parameters: maximum tumor diameter (R), exophytic ratio (E), nearest distance to the renal sinus (N), anterior‒posterior location (A), and relationship to the polar line (L). CSA represents the tumor contact surface area. The meta–analyses confirmed a strong correlation between R.E.N.A.L. and PADUA (*r* = 0.8607) and their efficacy in outcome prediction [11–16]. The CSA score has unique advantages because it can assess residual renal function after surgery [17–21]. However, all score systems rely on manual calculation by clinicians and are plagued by inefficiency, subjectivity, poor reproducibility, suboptimal accuracy, and limited clinical utility. Simplified newer alternatives created by scholars have failed to address these limitations [22–25]. Artificial intelligence (AI), which leverages automation, efficiency, and precision, has emerged as a transformative tool in RCC management, demonstrating promise in diagnosis, treatment planning, and prognosis [26–32]. At present, the exploration of R.E.N.A.L. and CSA scores generated by AI have been reported only individually [33, 34], and its efficacy needs to be further improved.

This study aims to develop and validate a CT-based automated kidney/tumor segmentation model, and on this basis, further construct the AI-R.E.N.A.L. score model (with the “A” parameter, which only provides qualitative classification, excluded from the original R.E.N.A.L. score) and AI-CSA score model. These models enable accurate prediction of perioperative outcomes of NSS in RCC patients, ultimately improving the objectivity of NSS preoperative assessment and the efficiency of clinical decision-making.

## Methods

### Ethical approval and data collection

This study was approved by the Institutional Ethics Committee of Fuzhou University Affiliated Provincial Hospital and Fujian Cancer Hospital, with approval numbers K2021–12–018 and SQ2023–107, respectively. Given that the data used in this study are retrospective, the ethics committee waived the requirement for informed consent. Retrospectively, RCC patients who underwent NSS were collected from two centers: Fuzhou University Affiliated Provincial Hospital (n1 cohort: January 2016–January 2025) and Fujian Cancer Hospital (n2 cohort: January 2023–January 2025). The inclusion criteria were as follows: 1) All patients underwent laparoscopic NSS, while open surgery and robotic-assisted NSS were excluded; 2)postoperative pathologically confirmed RCC; 3) complete contrast–enhanced arterial–phase CT imaging of the kidney and tumor; and 4) comprehensive clinical records available for analysis. The exclusion criteria were as follows: (1) CT images containing artifacts that interfere with score or model development and (2) the presence of multiple renal tumors (in a single or bilateral kidney).

### Manual score

The manual scoring of R.E.N.A.L. and CSA consists of two stages: First, the first round of scoring is conducted independently by 4 junior radiologists (with 3 to 5 years of clinical experience in RCC diagnosis); subsequently, one senior radiologist and one senior urologist (with 12 and 11 years of professional experience in RCC, respectively) independently reviewed the scores. Discrepancies in scores were resolved through team discussion. All the doctors involved in the manual scoring and image annotation were blinded to the clinical data to avoid bias. Manual R.E.N.A.L. scores were based on the scoring criteria established by Kutikov et al. [8] (Figure S1). The manual CSA was calculated via a spherical crown–like surface area formula: $\:\text{S}={\uppi\:}\text{R}\text{h}$, where R represents the tumor diameter and h denotes the depth of the tumor within the renal parenchyma.

### Developing and validating AI score models

#### Datasets and preprocessing

This project relies on the two-center datasets from Fuzhou University Affiliated Provincial Hospital and Fujian Cancer Hospital, and draws on the advanced technologies in medical image processing and artificial intelligence from the College of Physics and Information Engineering, Fuzhou University. Regarding the datasets, Cohort n1 includes 500 cases and Cohort n2 includes 50 cases. All datasets contain complete arterial-phase CT images with a resolution of 512 × 512. Raw DICOM files were converted to NIfTI format via Python scripts (based on the dicom2nifti library), with voxel sizes standardized and background noise cropped. Mimics Research 21.0 software was used for manual image annotation.

#### Development and validation of automated kidney/tumor segmentation models

**Fig. 1:**
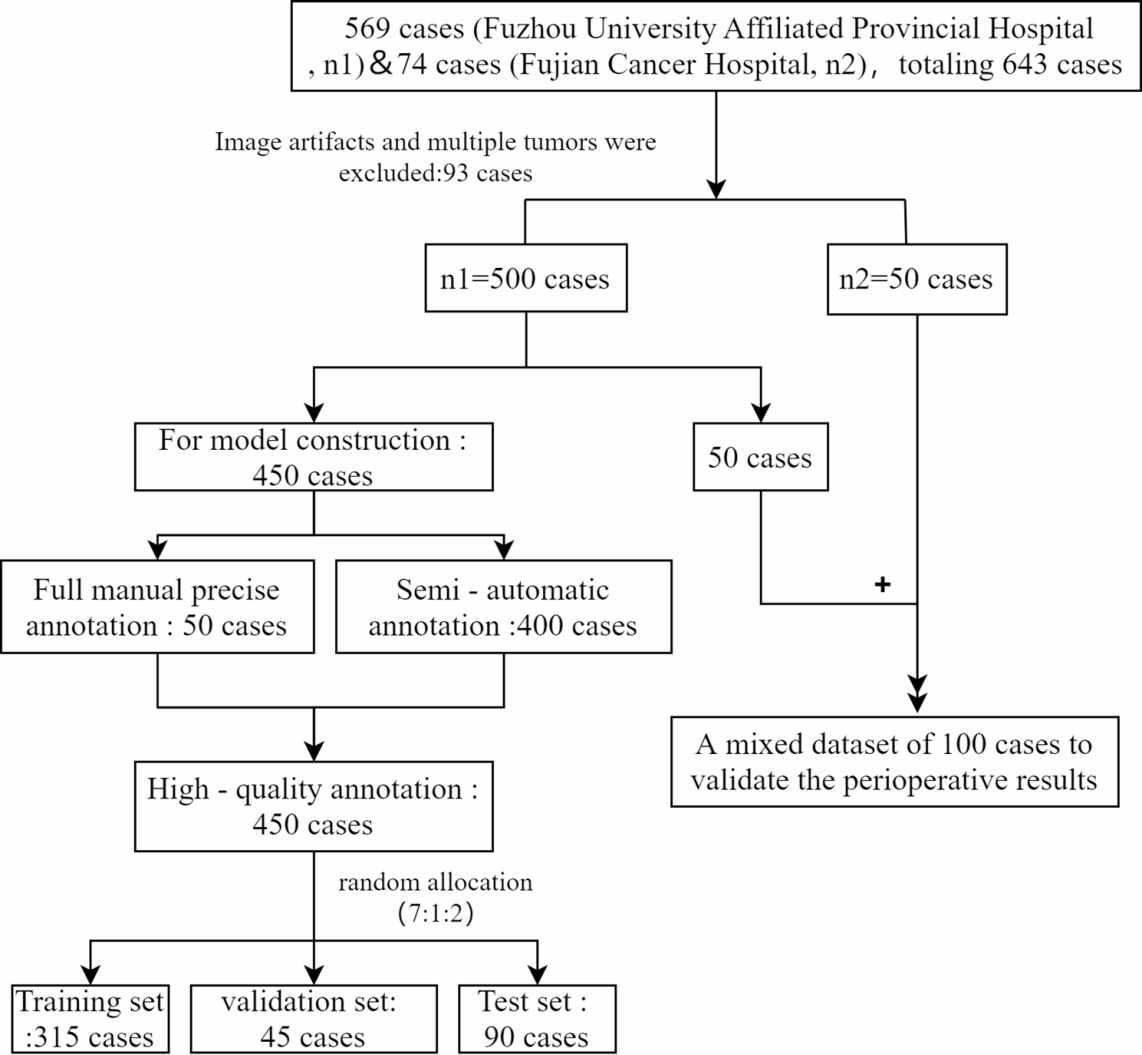
Data distribution flowchart

To address the issue of high costs associated with 3D imaging data annotation, this study adopts a phased annotation strategy of “full manual precise annotation - semi–automatic rough annotation - manual review iteration”: ① Full manual precise annotation phase: A senior radiologist and a senior urologist conduct full manual precise annotation on 50 cases of images (from n1), which are used as seed data to train the nnU –Net model; ② Semi–automatic rough annotation + manual review phase: The trained nnU–Net model is used to perform rough annotation on the remaining 400 cases of images (from n1). Subsequently, 4 junior radiologists revise the rough annotation results independently, which are then reviewed by one senior radiologist and one senior urologist. Through multiple rounds of iteration of “semi–automatic rough annotation - manual revision - expert review - model feedback”, 400 cases of semi–automatic annotation data that have undergone manual review are finally obtained; ③ Data usage differentiation: The high–quality annotation data used for model training include 50 cases of full manual annotation and 400 cases of semi–automatic annotation that have undergone manual review (450 cases in total); the 100 cases of data used for perioperative outcome prediction are the results of automatic annotation by the model. During model training, these 450 cases of high–quality annotation data are randomly allocated to the training set (315 cases), validation set (45 cases), and test set (90 cases) at a ratio of 7:1:2. Figure 1 shows the data distribution flowchart. The input of the training model is the pre–processed single–channel CT images, and the output is the binary segmentation results of the kidney and tumor. Prior to training, the data underwent three preprocessing steps: (1) spatial normalization—images were resampled to the median voxel size via trilinear interpolation, with labels resampled via nearest–neighbor interpolation to preserve anatomical boundaries; (2) intensity standardization—after adjusting the window width/level to 400 HU/30 HU, pixel intensities were normalized via Z–score transformation to standardize brightness across scans; and (3) data augmentation—random rotations (± 15°), elastic deformations, and scaling (0.8–1.2×)—were applied to enhance model generalization and reduce overfitting. The training was iterated for a total of 505 rounds, and overfitting was avoided through the early stop method (monitored by the internal validation set). Model performance for renal and tumor segmentation was evaluated via the Dice similarity coefficient (DSC), Normalized surface distancev(NSD) and 95th percentile Hausdorff distance (HD95).

#### Development and validation of the R.E.N.A.L. score model

Based on the segmentation results, the R.E.N.A.L. score model parameters were defined as follows: (1) R (maximum tumor diameter): calculated via 3D connected component analysis and the minimum bounding box method to measure the longest tumor dimension; (2) E (exophytic ratio): the renal parenchymal boundary was reconstructed to quantify the proportion of tumor volume protruding outside the kidney, defined as the exophytic volume divided by total tumor volume; and (3) N (nearest distance to the renal sinus): the renal sinus was segmented using a HU threshold (-110 to 0), and the minimum Euclidean distance between the tumor and sinus region was computed. (4) L (relationship to the polar line): Renal poles were localized via morphological analysis, and the proportion of tumor voxels crossing the polar line (connecting the upper and lower renal poles) was determined. (5) R.E.N.A.L. total score: Sum of the R, E, N, and L parameters. Notably, the “A” parameter was excluded from the score.

#### Development and validation of the CSA score model

The process of the CSA score model mainly includes the following steps: (1) Contact surface extraction: the marching cubes algorithm was applied to generate a triangular mesh representing the tumor–renal parenchyma contact surface from segmentation masks; (2) area calculation: on the basis of the vertex coordinates of the triangular mesh and voxel physical dimensions, the area of each triangle was computed and summed to obtain the total contact surface area; and (3) unit conversion: the result was converted from mm² to cm² by dividing by 100.

#### AI–R.E.N.A.L. And AI–CSA enable risk stratification of NSS-related perioperative outcomes

R.E.N.A.L. score risk stratification: low complexity (4–6 points), medium complexity (7–9 points) and high complexity (10–12 points). CSA score risk stratification was as follows: low-CSA group (CSA value < 20 cm²) and high-CSA group (CSA value ≥ 20 cm²). The evaluated outcomes included WIT, surgical duration, intraoperative blood loss, serum creatinine change, postoperative hospital stay, pathological T staging, and nuclear grade. WIT was defined as the duration from renal artery clamping to reperfusion. Surgical duration was calculated as the time from anesthesia induction to anesthesia termination. Postoperative serum creatinine changes were measured as the difference between day 1 postoperative and preoperative creatinine levels. The nuclear classification is based on the WHO/ISUP standard (Fifth Edition).

### Statistical analysis

Statistical analyses were performed via SPSS 27.0 and GraphPad Prism 9.5. Continuous variables are summarized as medians (interquartile ranges, IQRs), and categorical variables are summarized as frequencies (percentages). Data normality was tested via the Shapiro–Wilk test. Agreement between automated and manual scores was evaluated via the weighted Kappa coefficient. For risk stratification of NSS perioperative outcomes, the Kruskal–Wallis test and Mann–Whitney U test were used for group comparisons, with post hoc Dunn’s multiple comparisons test used to identify pairwise differences. Statistical significance was set at *P* < 0.05.

## Results

### Cohort characteristics and flowchart

**Fig. 2:**
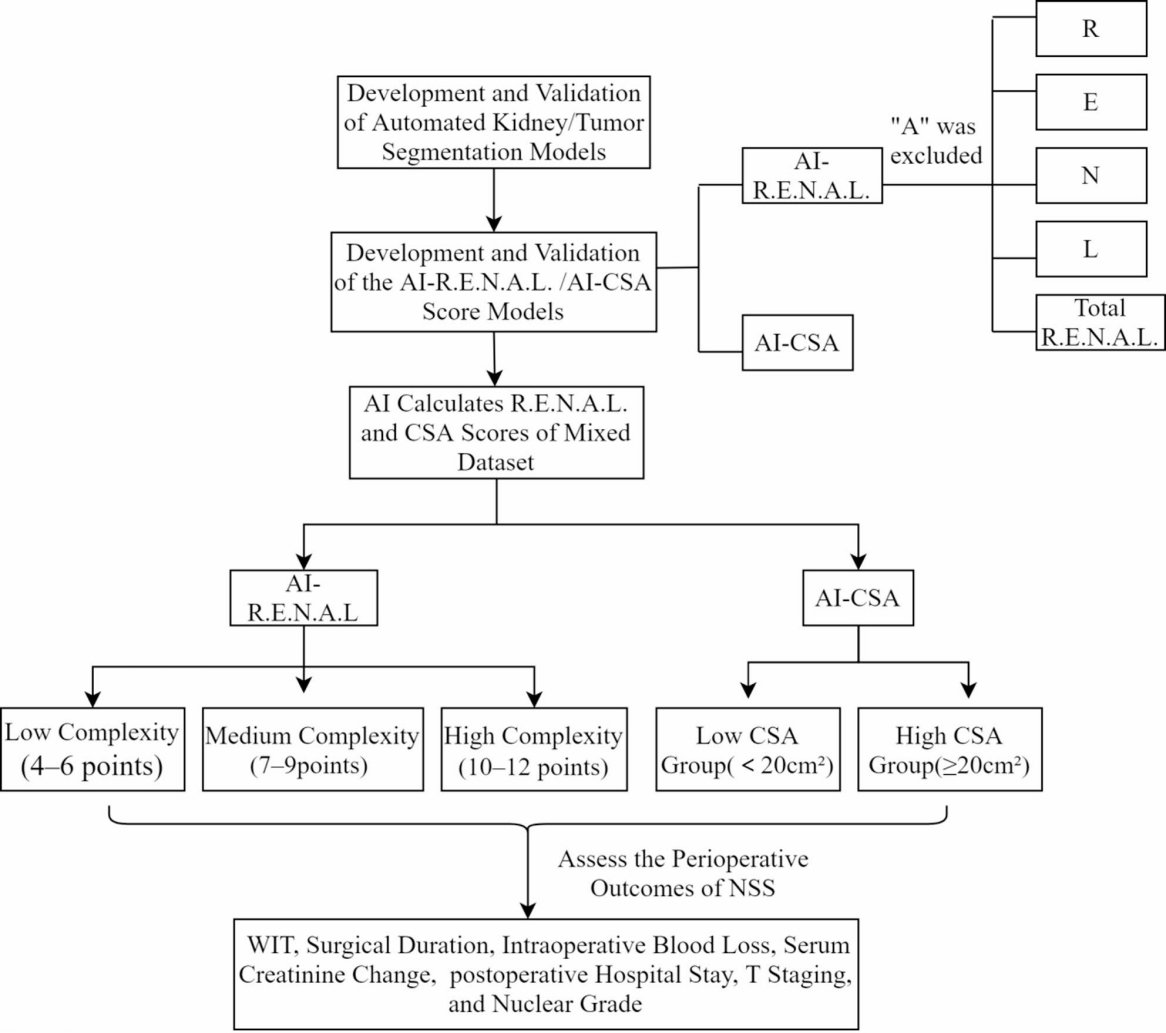
Flowchart of the technical route of this research

On the basis of the inclusion and exclusion criteria, a total of *N* = 550 patients were ultimately included in this study, consisting of 500 patients from Fuzhou University Affiliated Provincial Hospital (n1 cohort) and 50 patients from Fujian Cancer Hospital (n2 cohort). This real–world dataset included a cohort with a median age of 56 (46–66) years, comprising 341 males (62.0%) and 209 females (38%). The majority of cases (471, 85.64%) were detected incidentally during routine physical examinations. Hypertension and diabetes were present in 199 (36.18%) and 78 (18.18%) patients, respectively. The median values for preoperative serum creatinine, postoperative day 1 serum creatinine, and creatinine changes were 73 (60–82) µmol/L, 80 (66–97) µmol/L, and 9 (1–19) µmol/L, respectively. Preoperative prophylactic antibiotic use was recorded in 268 patients (48.73%). Pathological analysis revealed clear cell carcinoma as the predominant histotype (449 cases, 81.64%), with most tumors staged as T1a (428 cases, 77.82%) or nuclear grade 1/2 (82.55%). The remaining demographic and clinical characteristics are detailed in Table 1. Figure 2 shows the flowchart of the technical route of this research.

**Table 1:** Cohort characteristics

Demographic and clinical characteristics		*N* = 550
Gender - n(%)	Male	341(62.0%)
	Female	209(38.0%)
Age(yrs.)- Median (IQR)	-	56(46–66)
BMI(kg/m²)- Median (IQR)	-	24.11(22.03–26.10)
Symptom - n(%)	Incidentally detected	471(85.64%)
	Pain	27(4.91%)
	Hematuria	34(6.18%)
	Others	18(3.27%)
Hypertension - n(%)	Yes	199(36.18%)
	No	351(63.82%)
Diabetes - n(%)	Yes	78(18.18%)
	No	472(85.82%)
Preoperative serum creatinine(µmol/L)- Median (IQR)	-	73(60–82)
Day 1 postoperative serum creatinine(µmol/L)- Median (IQR)	-	80(66–97)
Serum creatinine change(µmol/L) - Median (IQR)	-	9(1–19)
Prophylactic use of antibiotics before the operation - n(%)	Yes	268(48.73%)
	No	282(51.27%)
Pathological types - n(%)	Clear cell carcinoma	449(81.64%)
	Papillary cell carcinoma	17(3.09%)
	Chromophobe cell carcinoma	34(6.18%)
	Others	50(9.09%)
T staging - n (%)	T1a	428(77.82%)
	Stage T1b and above	122(22.18%)
nuclear classification - n (%)	1/2	454(82.55%)
	3/4	96(17.45%)

IQR: Interquartile range

### Automated kidney and tumor segmentation models

**Fig. 3:**
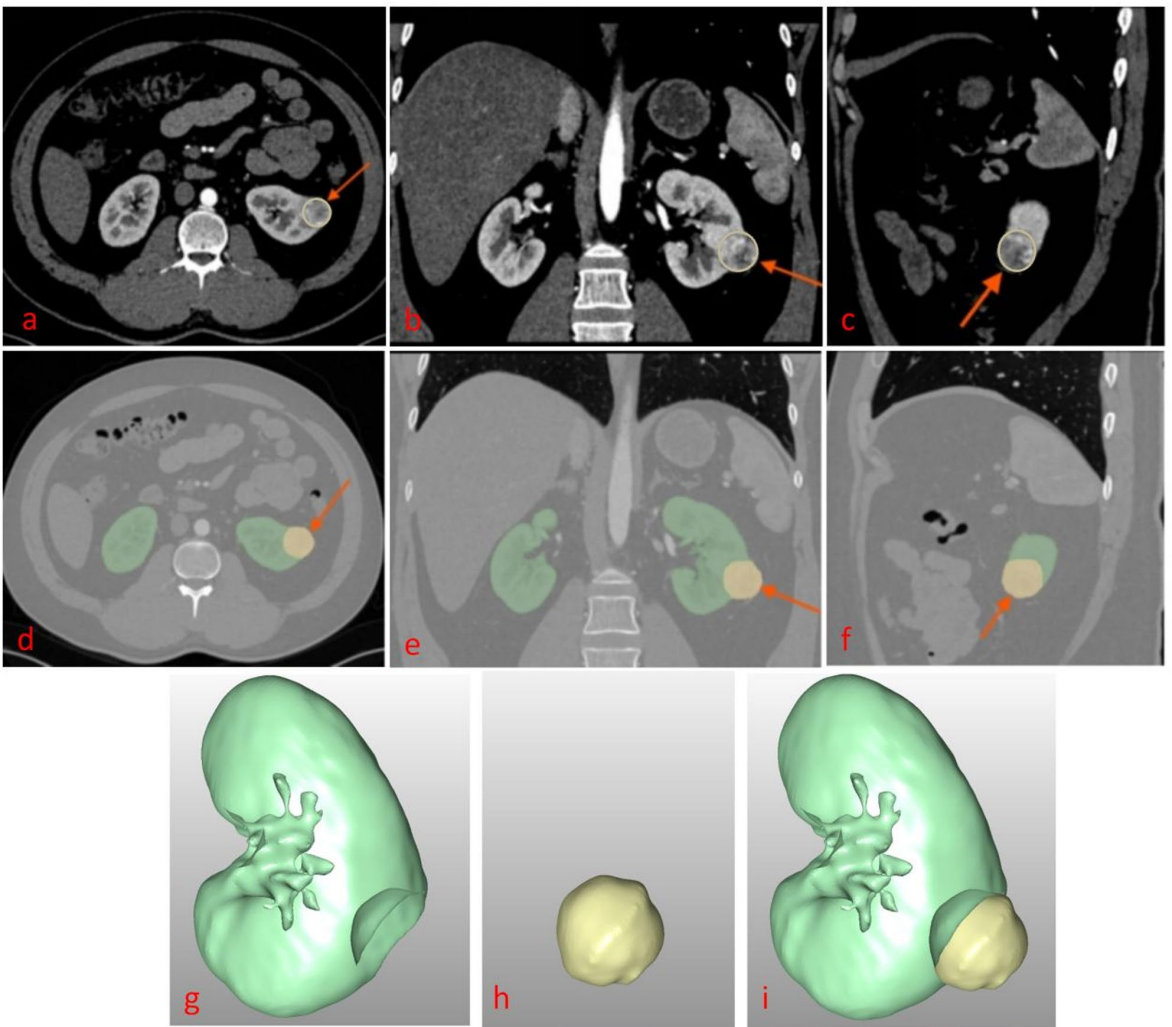
A 31-year-old male with incidentally detected left renal clear cell carcinoma (pathological stage T1a, grade 2). Panels **a**, **b**, and **c** show original axial, coronal, and sagittal CT arterial-phase images, respectively (red arrows: tumor). Panels **d**, **e**, and **f** depict corresponding axial, coronal, and sagittal views of AI-annotated kidney and tumor contours (red arrows: tumor). Panels **g**, **h**, and **i** present 3D reconstructed segmentation labels for the kidney, tumor, and their combined overlay, respectively

**Fig. 4:**
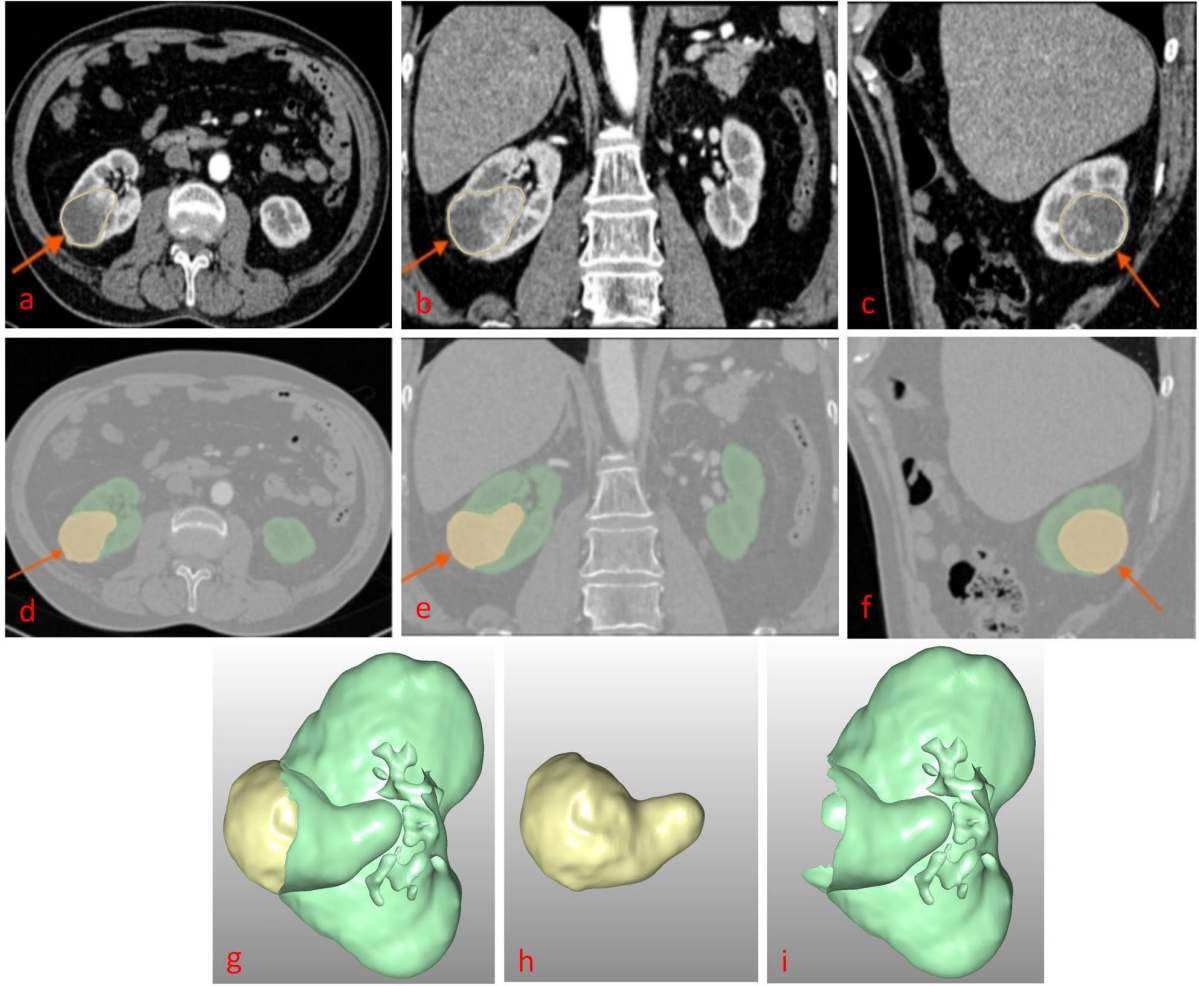
A 70-year-old male with incidentally detected right renal clear cell carcinoma (pathological stage T1b, grade 3). Panels **a**, **b**, and **c** display original axial, coronal, and sagittal CT arterial-phase images, respectively (red arrows: tumor). Panels **d**, **e**, and **f** show corresponding axial, coronal, and sagittal views of the AI-annotated kidney and tumor contours (red arrows: tumor). Panels **g**, **h**, and **i** present 3D reconstructed segmentation labels for the kidney, tumor, and their combined overlay, respectively

The DSCs values are 0.95 (for bilateral kidneys) and 0.80 (for tumors); The NSD values are 0.923 ± 0.082 (for bilateral kidneys) and 0.892 ± 0.096 (for tumors); the HD95 values are 9.78 ± 0.63 mm (for bilateral kidneys) and 12.65 ± 0.84 mm (for tumors). Figures 3 and 4 depict schematic visualizations of the segmentation results for two representative cases. Figure 3 illustrates a low–complexity case: both manual–calculated and AI–calculated R.E.N.A.L. (AI–R.E.N.A.L.) scores were $\:1+1+1+2=5$, with manually–calculated CSA (M–CSA) of 14.83 cm² and an AI–calculated CSA (AI–CSA) of 14.26 cm². Figure 4 shows a high–complexity case (irregular morphology with extensive necrosis and liquefaction): the manually calculated R.E.N.A.L. score (M–R.E.N.A.L.) was $\:2+3+3+3=11$, whereas the AI–R.E.N.A.L. $\:2+2+3+3=10$; the M–CSA and AI–CSA values were 67.86 cm² and 59.07 cm², respectively. Both cases demonstrated the excellent segmentation performance of the AI model, particularly in capturing anatomical details across varying levels of tumor complexity.

### Agreement validation between AI–calculated R.E.N.A.L./CSA scores and manual scores

Agreement for six parameters–R, E, N, L, R.E.N.A.L. total score and CSA were assessed via weighted kappa coefficients, and the detailed results are presented in Table 2. The median (IQR) of M–CSA was 18.56 (10.30–27.00) cm², whereas it was 14.17 (7.76–21.10) cm² for AI–CSA.

**Table 2:** Consistency verification of AI-R.E.N.A.L. score /AI-CSA score versus manual score

Manual VS AI (*n* = 90)	Weighted Kappa coefficients	Standard error	95% CI	*P* Value	Agreement
R	0.82	0.07	0.68–0.96	<0.01	Almost perfect
E	0.49	0.06	0.38–0.61	<0.01	Moderate
N	0.63	0.06	0.51–0.74	<0.01	Substantial
L	0.60	0.06	0.48–0.71	<0.01	Substantial
R.E.N.A.L.	0.65	0.04	0.57–0.72	<0.01	Substantial
CSA	0.69	0.03	0.63–0.75	<0.01	Substantial

AI : Artificial intelligence

CSA : Contact surface area

AI-R.E.N.A.L. : AI–calculated R.E.N.A.L

AI-CSA: AI–calculated CSA

### AI–R.E.N.A.L. And AI–CSA enable risk stratification of NSS-related perioperative outcomes

**Fig. 5:**
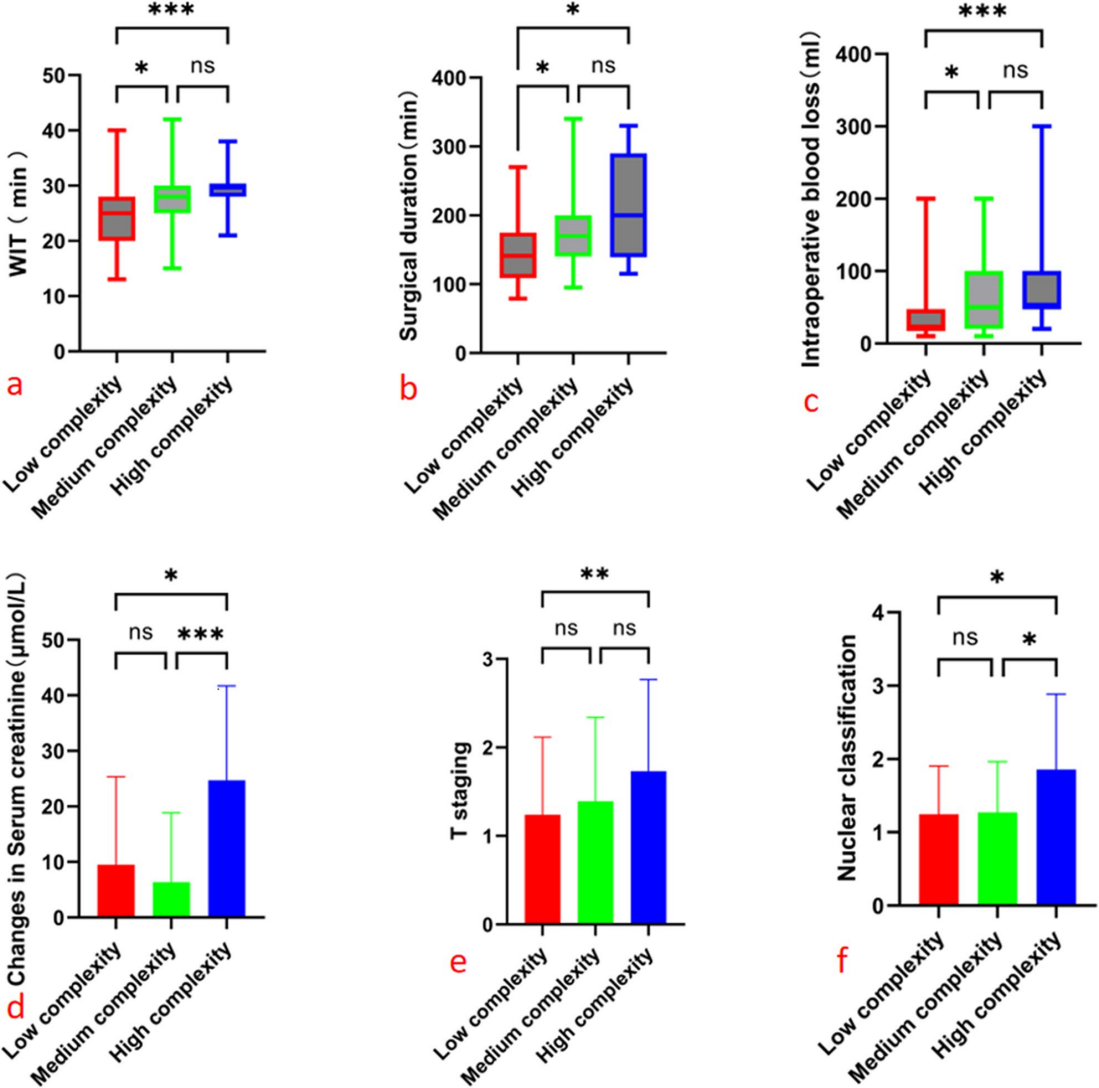
Statistical differences across risk stratification groups were evaluated via Dunn’s multiple comparisons test: (**a**) WIT, (**b**) surgical duration, (**c**) intraoperative blood loss, (**d**) serum creatinine change, (**e**) T - staging, (**f**) nuclear classification

On the basis of the previous findings, both AI–R.E.N.A.L. and AI–CSA scores showed substantial agreement with the manual assessments. Thus, AI–R.E.N.A.L. was directly applied to stratify risks into low-, moderate-, and high-complexity groups, predicting intergroup differences in WIT, operative duration, intraoperative blood loss, postoperative serum creatinine change, postoperative hospital stay, pathological stage, and nuclear grade (detailed results in Table 3; Fig. 5). Similarly, AI–CSA scores were directly used for risk stratification into low- and high-score groups, and the results are presented in Table 4.

**Table 3:** Risk stratification of perioperative outcomes for NSS by AI-R.E.N.A.L.

AI-*R*.E.*N*.A.L. (*N* = 100)	low complexity(4–6)	moderate complexity(7–9)	high complexity(10–12)	Kruskal-Wallis statistic	*P*
n(%)	46(46%)	39(39%)	15(15%)		
WIT (min) - Median(IQR)	25(20–28)	28(25–30)	30(28–30)	16.63	<0.01
Surgical duration (min) - Median (IQR)	141(109–175)	170(140–200)	200(139–290)	10.87	<0.01
Intraoperative blood loss (ml) - Median (IQR)	20(20–47)	50(20–100)	50(50–100)	18.30	<0.01
Postoperative serum creatinine change (µmol/L) - M (IQR)	8(4–18)	3(−2−13)	20(12–33)	13.86	<0.01
Postoperative hospital stay (day) - Median (IQR)	8(4–11)	8(5–10)	6(5–6)	4.51	0.10
T staging - Median	T1a	T1a	T1b	11.12	<0.01
Nuclear classification	2(2–2)	2(2–2)	2(2–3)	7.40	0.02

NSS : Nephron–sparing surgery

AI : Artificial intelligence

AI-R.E.N.A.L. : AI–calculated R.E.N.A.L

WIT : Warm ischemia time

IQR : Interquartile range

**Table 4:** Risk stratification of perioperative outcomes for NSS by AI-CSA

AI-CSA (*N* = 100)	Low score group(<20 cm²)	High score group(≥20 cm²)	Mann-Whitney U test-U value	*P*
N(%)	66(66%)	34(34%)		
WIT (min) - Median (IQR)	25(20–28)	30(26–30)	516.5	<0.01
Surgical duration (min) - Median (IQR)	142(124–175)	185(148–243)	647.5	<0.01
Intraoperative blood loss (ml) - Median (IQR)	30(20–50)	50(30–100)	680.0	<0.01
Serum creatinine change (µmol/L) - M (IQR)	7(0–17)	11(1–25)	720.5	0.16
Postoperative hospital stay (day) - Median (IQR)	8(4.75-11)	6.5(5–8)	1250.0	0.35
T staging - Median	T1a	T1a	682.5	<0.01
Nuclear classification	2	2	915.0	0.77

NSS : Nephron–sparing surgery

AI : Artificial intelligence

CSA : Contact surface area

AI–CSA : AI–calculated CSA

WIT : Warm ischemia time

IQR : Interquartile range

## Discussion

First, we successfully developed an automated CT–based segmentation model for kidneys and tumors, which demonstrated favorable performance compared with manual segmentation. On the basis of this model, we subsequently constructed AI–R.E.N.A.L. and the AI–CSA score models, both of which strongly agreed with the manual score. Finally, risk stratification of NSS perioperative outcomes via these models revealed that AI scores effectively predict WIT, surgical duration, intraoperative blood loss, postoperative serum creatinine change, pathological stage, and nuclear grade. Furthermore, we adopted a combined approach of manual annotation and semi–automatic annotation, which significantly improved the efficiency and accuracy of creating high-quality annotation data. The kidney/tumor segmentation model constructed accordingly exhibited favorable performance, with DSC, NSD, and HD95 comparable to the average results of the KiTS challenge [35].

For the AI–R.E.N.A.L. model, weighted Kappa coefficients for parameters R, E, N, L, and total R.E.N.A.L. scores were 0.82, 0.49, 0.63, 0.60, and 0.65, respectively, indicating almost perfect agreement for R and substantial agreement for N, L and total R.E.N.A.L. score and moderate agreement for E. These results exceed the clinical utility reported by Heller et al. [33]. The AI–CSA model showed strong agreement with M–CSA (Kappa = 0.69), which was comparable to the results of Wood et al. [34]. Notably, AI–CSA directly calculates the tumor–kidney contact surface via pixel–level analysis, avoiding the systematic bias of manual methods, which assume spherical tumor geometry and often overestimate M–CSA (median M–CSA: 18.56 [10.30–27.00] cm² vs. AI–CSA: 14.17 [7.76–21.10] cm²).

The AI-R.E.N.A.L. and AI-CSA scoring models were used for risk stratification of perioperative outcomes of nephron-sparing surgery (NSS). Specifically, the AI-R.E.N.A.L. score was stratified into low complexity (4–6 points), moderate complexity (7–9 points), and high complexity (10–12 points), with statistically significant differences (*P* < 0.05) observed in multiple key indicators between groups: the WIT was 25 min, 28 min, and 30 min in sequence, with longer WIT in the moderate/high-complexity groups than in the low-complexity group—this is consistent with reports in the literature [36, 37], reminding clinicians to control ischemia duration; the median surgical duration was 141 min, 170 min, and 200 min in sequence, with longer duration in the moderate/high-complexity groups, which is consistent with the findings of Wang et al. [38]; the median intraoperative blood loss was 20 ml in the low-complexity group and 50 ml in both the moderate and high-complexity groups, with more blood loss in the moderate/high-complexity groups—Styopushkin et al. [39] also confirmed the correlation between tumor complexity and bleeding risk, requiring enhanced risk prevention and control; the median postoperative serum creatinine change was 8 µmol/L, 3 µmol/L, and 20 µmol/L in sequence, with more significant elevation of postoperative creatinine in the high-complexity group, which echoes the finding in the literature [40] that “tumor complexity is positively correlated with serum creatinine change,” necessitating close follow-up of renal function; the proportion of pathological T1a stage was 90.48%, 76.32%, and 46.67% in sequence, with a lower proportion of low-stage cases in the high-complexity group; the proportion of nuclear grade 1/2 was 87.80%, 86.49%, and 57.14% in sequence, with higher nuclear grade in the high-complexity group, indicating higher invasiveness and recurrence risk [41]. However, there was no statistical difference in postoperative hospital stay, presumably due to all cases in this study being laparoscopic NSS (minimally invasive surgery reduces data variability). The AI-CSA score was stratified into the low-CSA group (< 20 cm²) and high-CSA group (≥ 20 cm²). Statistically significant differences (*P* < 0.05) were observed in the following indicators: WIT (25 min vs. 30 min), surgical duration (142 min vs. 185 min), intraoperative blood loss (30 ml vs. 50 ml), and proportion of pathological T1a stage (88.89% vs. 56.25%)—all indicating higher surgical difficulty and pathological risk in the high-CSA group—while no intergroup differences were found in serum creatinine change, postoperative hospital stay, or nuclear grade. Leslie et al. [17] also confirmed a positive correlation between the CSA score and warm ischemia time, surgical duration, blood loss, postoperative serum creatinine change, and pathological stage, which is consistent with the results of this study.

The findings of this study have potential for clinical translation, specifically including: ① Automated preoperative planning: The AI quickly completes kidney/tumor segmentation and calculation of R.E.N.A.L. and CSA scores, outputs tumor anatomical features and risk stratification, and assists surgeons in predicting surgical difficulty (e.g., WIT) and formulating personalized plans; ② Intraoperative real-time reference: Import the 3D segmentation model generated by AI into the laparoscopic navigation system to intuitively present the spatial relationship between the tumor and renal blood vessels/collecting system, reducing intraoperative anatomical identification bias; ③ Postoperative follow-up assistance: Based on the high-risk groups predicted by AI, automatically mark patients requiring focused postoperative reexamination and optimize follow-up frequency.

The AI-R.E.N.A.L. and AI-CSA scoring models developed in this study can effectively predict perioperative outcomes of NSS, and relevant studies in the field form a good complement to it: ① Some studies [42–44] focus on the refinement of tumor location (expanding on the “N” parameter of the R.E.N.A.L. score in this study) and found that tumors near the collecting system (< 4 mm) or renal hilum have higher nuclear grades and invasiveness, suggesting that the AI score can further integrate this feature to improve the recognition efficiency of highly invasive cases; ② Study [45] points out that the combined evaluation of tumor location and volume guides surgical approach selection, which is consistent with the logic of the AI in this study quantifying tumor size (R parameter) and location-related features (N, L parameters), and provides a direction for incorporating volume-derived indicators into the model; ③ Rosiello et al. [46] confirmed that the R.E.N.A.L., PADUA, and SPARE scores have comparable predictive efficacy for NSS perioperative outcomes, indicating that the AI automation framework of this study can be extended to other scoring systems; ④ Saitta et al. [47] found that NSS for patients with pT3aN0M0 renal cell carcinoma has both oncological safety and renal function preservation benefits, while the AI score can accurately identify such high-risk and high-complexity tumors (e.g., high CSA value, near renal hilum), which can assist in screening complex cases suitable for NSS; ⑤ Crocerossa et al. [48] clarified that early renal function change is a strong predictor of renal function decline 1 year after NSS, and the AI score in this study has been able to effectively predict key renal function-related indicators such as postoperative serum creatinine change (*P* < 0.05), which echoes this finding.

While this study achieved meaningful achievements, several limitations should be acknowledged. First, to avoid confounding segmentation and scoring of multiple tumors, the cohort excluded patients with multiple renal tumors, and future studies could include these populations to expand generalizability. Second, a small proportion of tumors smaller than 1 cm were under–segmented, and a minor subset of renal cysts were misclassified as tumors and erroneously segmented; future studies should further improve segmentation precision. Third, the analysis was limited to NSS data, and future research may explore perioperative outcomes in patients undergoing radical nephrectomy. Fourth, the model validation is dominated by internal evaluation—although 50 cases of external data from Cohort n2 have been included, this scale has not met the sample size requirement for true multi-center validation, and we will further expand the external institutional dataset for validation in the future. Fifth, the agreement between the AI-calculated E score and manually calculated E score remains weak, which needs further optimization and improvemen. Sixth, parameters such as WIT, surgical duration, intraoperative blood loss, and postoperative serum creatinine change may have potential bias influenced by confounding factors including surgeons’ surgical experience and patients’ underlying comorbidities (e.g., hypertension, diabetes mellitus). In the future, we will further expand the sample size and increase multicenter data to reduce the impact of such bias on the results.

## Conclusions

In summary, this study successfully developed a CT-based AI score model for RCC, which effectively performs risk stratification for NSS-related perioperative outcomes. The model demonstrates high efficiency and accuracy, featuring both robust automated kidney/tumor segmentation and precise prediction of key metrics, including WIT, surgical duration, intraoperative blood loss, postoperative serum creatinine change, pathological T stage, and nuclear grade. These capabilities have significantly increased the objectivity and accuracy of preoperative assessment, as well as the efficiency of clinical decision–making. They have reduced the previously required several hours of manual labor to just approximately ten minutes, highlighting their potential for clinical application. With advancements in AI technology, future research may explore more complex scenarios, such as radical nephrectomy and multifocal renal cancer, to enable AI to comprehensively support the entire workflow of renal cancer diagnosis and treatment.

## Supplementary Information

Below is the link to the electronic supplementary material.

Supplementary Material 1

## Data Availability

The datasets used and/or analyzed during the current study are available from the corresponding author upon reasonable request.

## Abbreviations

AI: Artificial intelligence
CSA: Contact surface area
RCC: Renal cell carcinoma
NSS: Nephron–sparing surgery
IQR: Interquartile range
DSC: Dice similarity coefficient
NSD: Normalized surface distance
HD95: 95th percentile Hausdorff distance
WIT: Warm ischemia time
M–CSA: Manually–calculated CSA
AI–CSA: AI–calculated CSA
M–R.E.N.A.L.: Manually–calculated R.E.N.A.L
AI–R.E.N.A.L.: AI–calculated R.E.N.A.L
