# Supplementary figures and images for: CT–based AI score associates with perioperative outcomes in nephron–sparing surgery for renal cell carcinoma
Source: Cancer Imaging. 2025 Dec 29;25:139. doi: 10.1186/s40644-025-00961-2 (PMC12751408; doi:10.1186/s40644-025-00961-2)

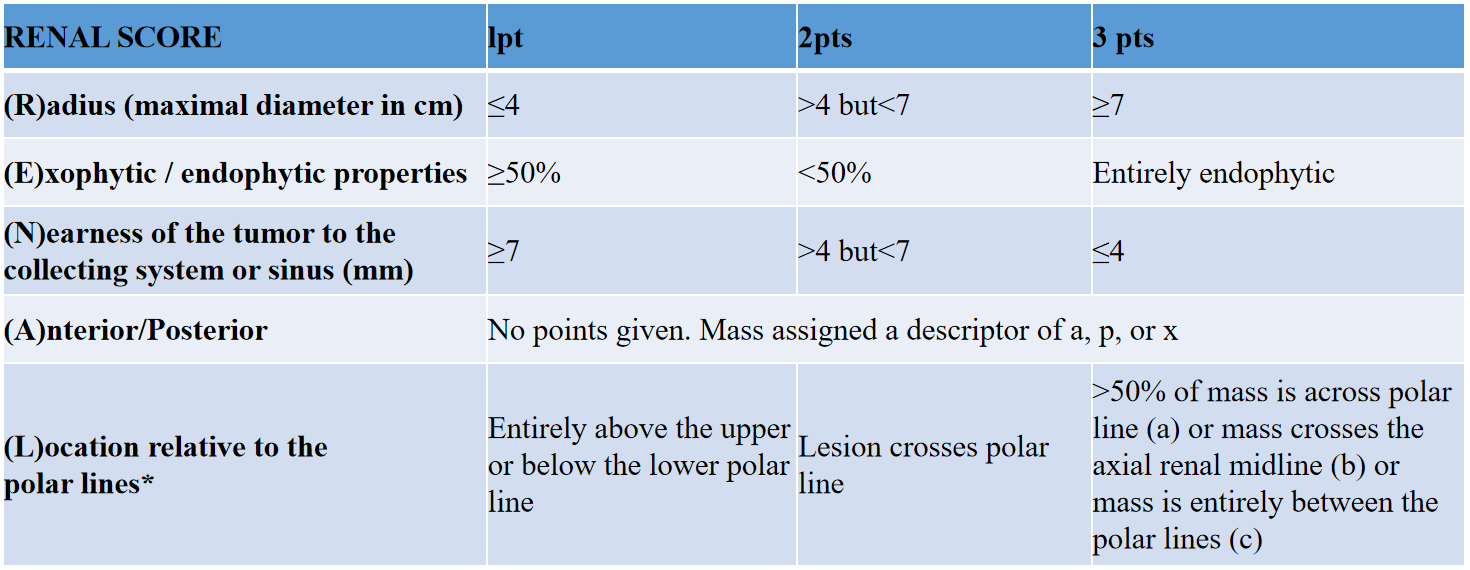

Supplement: Supplementary file 1 — Supplementary Material 1 [file 40644_2025_961_MOESM1_ESM.tif]
